# Assessing the Practice of Birth Defect Registration at Addis Ababa Health Facilities

**DOI:** 10.4314/ejhs.v31i3.26

**Published:** 2021-05

**Authors:** Mekonen Eshete, Fikre Abate, Berhane Abera, Abiye Hailu, Yohannes Demissie, Peter Mossey, Azeez Butali

**Affiliations:** 1 Surgical Department, School of Medicine, College of Health Sciences, Addis Ababa University, Addis Ababa, Ethiopia; 2 Plastic and Reconstructive Surgery Department Yekatit 12 Hospital Medical College, Addis Ababa, Ethiopia; 3 Department of Orthodontics, University of Dundee, Scotland, UK; 4 Department of Oral Pathology, Radiology and Medicine, College of Dentistry, University of Iowa, Iowa City, IA. U.S.A.

**Keywords:** Birth defect, Registration, surveillance

## Abstract

**Background:**

Birth defects are conditions that exist at birth and cause structural changes in one or more parts of the body. In order to plan proper management and design preventive activities of these conditions, accurate tracking, registration and analyses of the registered data are important. We assessed the practice of birth defect registration at Addis Ababa health facilities.

**Methods:**

We retrospectively checked the existence of a separate birth defect registry book and assessed the delivery room registration book for completeness in registering birth defects. We also assessed the total number of birth defects registered during 2010–2015.

**Results:**

We assessed the practice of birth defect registration at 37 delivery service providing health facilities in Addis Ababa, 20 public and 17 private institutions. Of the 37 health institutions assessed, 23 registered birth defects (3 of them used a separate birth defect registry books, and 20 used a regular registration book to register birth defects). The remaining 14 did not register any congenital anomaly. Of the institutions that do not register congenital anomalies, 10 are private and four are public.

**Conclusion:**

Only three delivery providing health facilities had a dedicated birth defect registry book which is close to ideal for a birth defect registration. There is a need for others to do the same until an electronic birth defect registration is established. This registration will serve as a resource for clinical governance and studies into quality of life, quality of care, etiology and prevention.

## Introduction

Birth defects are structural or functional/developmental abnormalities present at birth. They can cause physical disability, intellectual and developmental disability, and other health problems. Birth defects are a major contributor to infant mortality, and the care might cost billions of dollars (1) Major structural or functional birth defects affect approximately 3% of births in the United States. The causes of most major birth defects are unknown (2). Birth defects are the fourth leading causes of neonatal death worldwide. As it was shown by C. P. Howson and B. Modell the number of children born with major birth defects is around 7.9 million (6% of births) per year globally. Birth defects contribute significantly to neonatal deaths. For instance, in 2010, about 9% of all neonatal deaths in 193 countries around the world were due to birth defects(3)

Birth defects cause significant burden to the victim and to society. They are the main cause of early abortion, stillbirth, perinatal and infant death and birth disability. This is definitely a public issue of population quality and population health. Birth defects are considered as a curse in many developing countries, including Ethiopia, leading to stigmatization and effect on quality of life. (4)

A systematic and well-planned data registry is very important to analyze the extent of the problem and its burden on the society. In order to properly manage birth defects and plan preventive strategy, it is very important to establish a standard birth defect registry system. It can also be a very efficient and cost-effective means of identifying eligible children for treatment and providing timely referral to specialized services (5). Due to the absence of a systematic birth defect registry system and a significant proportion of unattended delivery, the magnitude of birth defects and their impact on the community and the health system in Ethiopia remains unknown. In order to continue the conversation and advocate for the establishment of a birth defects registry in Ethiopia, we conducted a survey to assess the practice of birth defect registry system at Addis Ababa delivery service providing health facilities.

The objective of this surveillance was to assess how organised and comprehensive registration of birth defects is at Addis Ababa delivery service providing health facilities.

## Materials and Methods

This study is part of the larger Ethiopian genetics study entitled “Investigating the role of genetics and environmental factors in the occurrence of orofacial clefts in the Ethiopian population” for which ethical approval was obtained from the Institutional Review Board, College of Health Sciences, Addis Ababa University (IRB approval number: 003/10/surg) and have been renewed every year. In addition, we obtained written permission from Addis Ababa City Administration Health Bureau (5/01/2007 E.C) and retrospectively assessed the Birth defect registry system at 37 delivery service providing health facilities in Addis Ababa. We collected the following information using open questions and observation:

The existent of a separate birth defect registry book

If training was provided on birth defect registry

If, after registering a birth defect, is there an onward referral to a specialized center

## Results

We evaluated the practice of birth defect registration at 37 delivery service providing health facilities in Addis Ababa, 20 public and 17 privates. All the 17 private health facilities are general hospitals and the public health facilities are five referral hospitals and the rest are health centers. These were the main delivery service providing institutions in Addis Ababa when we did the survey. Of the 37 health facilities, 23 registered birth defects (3 of them used a separate birth defect registry books, while 20 used the delivery ward registration book to register birth defects). The remaining 14 have not registered any congenital anomaly. Of the institutions that did not register congenital anomalies, 10 are private and four are public. Only three of the health facilities have midwives who have some orientation and training on birth defects and the importance registration, all the three were from the private hospitals.

The total number of live births at the study health facilities from 2010–2015 was 156,272, and there were 3215 (2.06%) still births. During this time, 543(0.35%) birth defects of different types were registered since there is only one column in the delivery ward registry book, detailed information about the type of birth defect and its severity was not registered. Of the children born with congenital anomalies registered, 196(36.1%) were still born.

We got a response from few delivery services providing midwives who were working at the health centers. These responses include “I did not register a birth defect because I did not know what it is called, so I just told them to go to Tikur Anbessa Hospital”. Tikur Anbessa Hospital is the largest pediatrics referral center for the country. The others said, “I did not know what it is called, so I just wrote congenital anomaly and told them to go to Tikur Anbessa Hospital”. Some said, “I delivered a child with cleft lip and referred to Yekatit 12 Hospital because I know they treat cleft patients”.

## Discussion

In addition to being a health and financial burden to communities, birth defects can also be a source of psychosocial problem to the patient and patient's family. Birth defects are considered as a curse in many developing countries including Ethiopia. Proper birth defect registry and surveillance is indispensable to address this issue. The findings of our study indicated that there is no established system of birth defect registration at health facilities in Addis Ababa, the capital city of Ethiopia. We feel it is the same in other parts of the country. The Sixty-third World Health Assembly, in 2010 recognized the importance of birth defects as a cause of stillbirths and neonatal mortality. During this same time, it was made clear that the lack, or inadequacy of birth defect registration systems and inaccurate records of the causes of death, in developing countries became a barrier to estimating the burden of public health problems associated to birth defects in this part of the world(6). Ethiopia's National Health Care Quality Strategy for 2016–2020 placed Maternal, Newborn and Child Health as a priority. The goals set were to reduce the maternal mortality ratio (MMR) from 412 to 199 per 100,000 live births by 2020; to reduce the neonatal mortality rate (NMR) from 28 to 10 per 1,000 live births by 2020 and reduce stillbirth rate from 18 to 10 per 1000 births by 2020(7).

Birth defects play a major role in neonatal mortality and stillbirth; therefore, proper birth defect registry system is crucial to know the magnitude of birth defect and plan a preventative mechanism.

The 0.35% birth defect report found in this study is a clear underestimate of birth defect at the studied institutions. As stated by Christianson and Modell, in 2004 (8) there is a scarcity of data on the birth prevalence of birth defects in middle- and low-income countries. The contributing factors for the underestimation of birth defects in these regions were: constrained diagnostic capability, poor health related statistics, lack of birth defect surveillance and registries and reliance on hospital-based rather than population-based studies.

The availability of accurate information is vital in order to take decisions on prevention and control of birth defects. It is also important to provide care and support to individuals affected by birth defects. The sixty third world health assembly urged member states to develop and strengthen registration and surveillance systems for birth defects within the framework of national health information systems(6).

Every year, around 3–6% of infants or approximately 8 million are born with a serious birth defect worldwide the majority, 90% of these infants are born in low- and middle-income countries and the impact of birth defects is mainly severe in these countries(9). Of the affected infants born annually, at least 1 in 3 die before age 5 and another 1 in 3 survive with significant disability. A new born with birth defects in a low- and middle-income countries is at much higher risk of dying than a similarly affected newborn in a high-income country This complication could be minimized by early identification of infants born with birth defects and providing support. Birth defect registries provide a useful means for early identification of infants born with birth defects which is very important for early intervention. Early intervention prevents secondary disabilities that results from primary condition(5).

Of the children born with congenital anomalies registered, 196(36.1%) were still born. This indicates that birth defect is associated with high still birth. Since there is no proper registration and follow-up on those who were born alive with congenital anomalies, it is not known whether they survive or not. This has a negative impact in fulfilling the Sustainable Development Goals 3.2 (“by 2030, end preventable deaths of newborns and children under 5 years of age, reduce neonatal mortality to at least as low as 12 per 1000 live births and under-5 mortality to at least as low as 25 per 1000 live births”) (10).

Some of the midwives practicing at the study institutions reported that they cannot indicate the type of birth defect they have seen, because they do not have training on birth defects. This has a negative impact on early referral to a specialized center to receive appropriate care. The importance of inclusion training about birth defects in the training of health professionals specially those providing delivery service and working with neonates is obvious. The Sixty-third World Health Assembly urges member states to raise awareness among all relevant stakeholders, including government officials, health professionals, civil society and the public, about the importance of newborn screening programs and their role in identifying infants born with congenital birth defects(6). To our knowledge, new born screening program is not part of the Ethiopian health care system yet.

The nonexistence of a systematic birth defect registration not only contributes to the poor care and support of the victims, it also led to the nonexistence of data on the burden of congenital anomalies on the society. Congenital anomalies account for 25.3–38.8 million disability-adjusted life-years (DALYs) worldwide(11, 12) There is no doubt that it will be much more in low- and middle-income countries like Ethiopia where there is no proper registry and care of children born with birth defects. The WHO systematic analysis for the Global Burden of Disease Study 2010 reports that birth defects rank 17^th^ in causes of disease burden (12).

In conclusion, Ethiopia is the second most populace country in Africa next to Nigeria. There is no established birth defect registration system in Ethiopia. Since there is no established training on birth defects, the delivery ward staff have a problem in registering birth defects. We recommend a proper birth defect registry and surveillance for Addis Ababa and for the country. This is a huge task which requires workforce training, developing a birth defect registration strategy and resource allocation. This study showed that birth defect is a major contributor for stillbirth. Therefore, we recommend to initiate a preventative activity to reduce birth defects which will contribute to the reduction of stillbirths. We also recommend to initiate new born screening programs which have a huge role in early identification of birth defects
